# A Novel Hemocyte-Specific Small Protein Participates in White Spot Syndrome Virus Infection via Binding to Viral Envelope Protein

**DOI:** 10.3390/v15010227

**Published:** 2023-01-13

**Authors:** Mingzhe Sun, Shihao Li, Yang Yu, Xiaojun Zhang, Fuhua Li

**Affiliations:** 1 Chinese Academy of Sciences (CAS) and Shandong Province Key Laboratory of Experimental Marine Biology, Institute of Oceanology, Chinese Academy of Sciences, Qingdao 266071, China; 2 Laboratory for Marine Biology and Biotechnology, Qingdao National Laboratory for Marine Science and Technology, Qingdao 266237, China; 3 Center for Ocean Mega-Science, Chinese Academy of Sciences, Qingdao 266071, China; 4 The Innovation of Seed Design, Chinese Academy of Sciences, Wuhan 430072, China

**Keywords:** a novel small protein, hemocyte, WSSV infection, shrimp

## Abstract

Hemocytes are essential components of the immune system against invading pathogens in shrimp. Many uncharacterized transcripts exist in hemocytes but the knowledge of them is very limited. In the present study, we identified a novel small protein from the uncharacterized transcripts in hemocytes of *Litopenaeus vannamei*. This transcript was specifically expressed in hemocytes and encoded a novel secretory protein, which was designated as hemocyte-specific small protein (LvHSSP). The expression level of *LvHSSP* was significantly up-regulated in the hemocytes of shrimp infected with white spot syndrome virus (WSSV). After knockdown of *LvHSSP* by RNA interference, the WSSV copy number in shrimp decreased significantly. Conversely, WSSV copy number increased in shrimp when they were infected by WSSV after incubation with recombinant LvHSSP protein. These results suggested that LvHSSP might promote viral infection in shrimp. Immunocytochemical assay showed that the recombinant LvHSSP protein was located on the membrane of hemocytes. Co-IP results showed that LvHSSP could interact with VP26, the main envelope protein of WSSV, suggesting that LvHSSP might mediate WSSV adhesion and entry into host cells by binding to viral envelope protein. Meanwhile, the total hemocyte counts were significantly decreased after *LvHSSP* knockdown while increased after supplementing with recombinant LvHSSP protein, supporting the idea of hemocytes as the carrier for systemic dissemination of WSSV. This study reported a novel small protein in hemocytes, which modulated the viral infection in shrimp. Our results will enrich the knowledge of invertebrate innate immunity and provide a new field in the study of hemocyte function.

## 1. Introduction

Shrimp products are known to be a significant source of aquatic animal protein [1]. Unfortunately, the industry has been plagued by outbreaks of bacterial or viral diseases [2]. White spot syndrome virus (WSSV) is one of the most prevalent, widespread, and lethal viruses and leads to serious economic loss in shrimp aquaculture worldwide [3,4]. Understanding shrimp antiviral immunity will be helpful to control the diseases. Like other invertebrates, the shrimp immune defense system lacks the adaptive immune system and relies only on innate immunity [5]. The circulatory fluid in shrimp, named hemolymph, is analogous to blood in vertebrates, which has also been proven to be vital to antimicrobial defense [6]. The hemolymph contains immune cells termed hemocytes, which could defend against invading pathogens through cellular and humoral immune responses including phagocytosis, apoptosis, coagulation, melanization, and production of antimicrobial peptides (AMPs), etc. [1].

In shrimp, hemolymph or hemocytes contain various proteins that exhibit immune defense ability to exclude pathogens. AMPs are important humoral immune effectors in shrimp, and hemocytes are the main source of AMP production [7]. Many isoforms of AMPs exhibited antiviral functions against WSSV [8,9]. Transglutaminase (TGase) released from hemocytes initiated the hemolymph coagulation by mediating the cross-linking aggregates of clotting proteins (CPs) [10,11]. Failure of coagulation induced by WSSV infection is beneficial for viral proliferation [12,13]. Moreover, as the main protein component of hemolymph, hemocyanin typically represents up to 95% of the total amount of hemolymph protein [14,15], which can inhibit the proliferation of WSSV in shrimp [16]. Some small fragments sourced from hemocyanin can attenuate the virus replication and cancer proliferation [17,18,19], which provided a new field for exploring active protein components.

Nowadays, with the improvements in bioinformatics and high-throughput technologies, several small proteins have been identified from the previously uncharacterized transcripts in crustacean, which exhibit important functions in immunity. A novel AMP, PcnAMP, from *Procambarus clarkia* exhibited a wide spectrum of antimicrobial activity [20]. A small open reading frame encoded peptide, MjRPS27, from *Marsupenaeus japonicus* showed antiviral activity by activating the NF-κB pathway [21]. Actually, numerous transcripts without annotation existed in the transcriptome of shrimp [22], but knowledge about the functions of these transcripts is very limited. In the present study, a novel small protein specifically expressed in hemocytes of the Pacific white shrimp *L. vannamei*, designated as LvHSSP, was studied due to its significant up-regulation in hemocytes when shrimp was challenged by WSSV. Its role in WSSV infection was clarified by RNA interference, recombinant protein treatment, co-immunoprecipitation (Co-IP), and immunocytochemical assay. To our knowledge, this is the first time to identify the function of a novel small protein during WSSV infection. The data will provide new insights into the innate immunity of hemocytes in crustaceans.

## 2. Materials and Methods

### 2.1. Experimental Animals, Viral Challenge and Tissue Collection

Healthy Pacific white shrimp cultured in our lab, with a body weight of 5.21 ± 0.33 g, were used for tissue distribution analysis, WSSV challenge, and RNA interference experiments. Before experiments, shrimp were reared in air-pumped circulating seawater at 25 ± 1 °C and fed with commercial food pellets for about a week. Shrimp were selected randomly for each experiment.

Hemolymph was collected from the ventral sinus located at the first abdominal segment using a syringe with an equal volume of precooled anticoagulant solution (115 mmol L^−1^ glucose, 27 mmol L^−1^ sodium citrate, 336 mmol L^−1^ NaCl, 9 mmol L^−1^ EDTA×Na_2_×2H_2_O, pH 7.4) [23]. Hemocytes were immediately collected by centrifugation at 1000× *g*, 4 °C, for 5 min. Different tissues including lymphoid organ, hepatopancreas, gills, intestine, epidermis, muscle, stomach, and heart were dissected from fifteen individuals, and each tissue from five individuals was put together as one sample. Three biological replicates were prepared for each tissue. The samples were preserved in liquid nitrogen for gene expression analysis.

To examine the expression pattern of *LvHSSP* in shrimp after WSSV challenge, shrimp were randomly divided into two groups (90 individuals in each group). For WSSV challenge, the virus particles were prepared according to the method described by Sun et al. [24]. The WSSV particles were diluted in sterilized phosphate-buffered saline (PBS) at a final concentration of 200 copies µL^−1^ and 10 μL was injected into each shrimp at the III and IV abdominal segments in the WSSV group. An equal volume of PBS was injected into each shrimp in the PBS group. At 0, 3, 6, 12, 24, and 48 h post-WSSV infection (hpi), the hemocytes from 15 individuals in each group were collected at each time point as described above for quantifying the mRNA expression levels. Hemocytes from five individuals were put together as one sample and three biological replicates were prepared.

### 2.2. RNA Extraction, cDNA Synthesis, Cloning and Sequence Analysis of LvHSSP

The total RNA was extracted by TRIzol reagent (Takara, Kyoto, Japan) and the cDNA template was synthesized using the PrimeScript RT Reagent Kit (Takara, Kyoto, Japan) with random primers according to the manufacturer’s protocols. Two specific primers LvHSSP-1F and LvHSSP-1R (Table S1) were designed to amplify and validate the nucleotide sequence of *LvHSSP* from the genome and transcriptome data [22]. Premix Ex Taq Hot Start version (TaKaRa, Kyoto, Japan) was used to amplify the gene. After the quality was assessed by electrophoresis on 1% agarose gel, the specific product was purified using the Gel Extraction Kit (Omega, Norcross, GA, USA), cloned into the pMD19-T vector (TaKaRa, Kyoto, Japan) and transformed into DH5α competent cells (TransGen Biotech, Beijing, China) for sequencing.

The complete ORF and amino acid sequence of *LvHSSP* was deduced using ORF finder (https://www.ncbi.nlm.nih.gov/orffinder/, accessed on 2 June 2022). Conserved protein domains were predicted with SMART (http://smart.embl-heidelberg.de/, accessed on 2 June 2022) and InterPro (http://www.ebi.ac.uk/interpro/, accessed on 2 June 2022). The theoretical isoelectric point (pI) and molecular weight (Mw) were calculated using ExPASy (https://web.expasy.org/compute_pi/, accessed on 7 June 2022).

### 2.3. Tissue Distribution and Detection of LvHSSP mRNA Expression

Semiquantitative RT-PCR was conducted to analyze the distribution of *LvHSSP* among different shrimp tissues using the primers LvHSSP-qF and LvHSSP-qR (Table S1). The amount of cDNA templates from shrimp tissues was quantified using an internal reference gene following the PCR condition described below: denaturation at 94 °C for 2 min; 30 cycles of 94 °C for 20 s, 56 °C for 20 s, and 72 °C for 20 s. *18S rRNA* (GenBank No. EU920969) was used as an internal control. The PCR products were detected by electrophoresis on 2% agarose gel.

Three hemocyte subpopulations in shrimp were isolated and their differentially expressed genes were identified using de novo transcriptome sequencing [25]. The expression level of *LvHSSP* (Unigene0002152 in this de novo transcriptome sequencing data) among three subpopulations was analyzed based on the RPKM value, and the significance between two subpopulations was characterized according to the *p*-value and false discovery rate (FDR).

Quantitative real-time RCR (qPCR) was used to analyze the expression patterns of *LvHSSP* at different time points after WSSV challenge with primers LvHSSP-qF and LvHSSP-qR (Table S1). *18S rRNA* was employed as an internal control for cDNA normalization. The PCR product was denatured to produce a melting curve to check the specificity of the PCR product.

### 2.4. RNA Interference Assay

The method for synthesis of double-stranded RNA (dsRNA) and RNAi assay was the same as described previously [26]. Briefly, A pair of primers with T7 promoter sequence, LvHSSP-dsF and LvHSSP-dsR (Table S1), were designed to amplify the fragment of the *LvHSSP* gene. The fragment of enhanced green fluorescent protein (EGFP) gene based on the pEGFP-N1 plasmid was cloned with primers of EGFP-dsF and EGFP-dsR (Table S1) for dsRNA synthesis. The PCR products were assessed by electrophoresis on 1% agarose gel and purified using the MiniBEST Fragment Purification Kit (Takara, Kyoto, Japan). The TranscriptAid T7 High Yield Transcription Kit (Thermo Fisher Scientific, Waltham, MA, USA) was used to synthesize the corresponding dsRNA from the purified products. Redundant single-strand RNA was digested by RNaseA (Takara, Kyoto, Japan). The concentration and quality of synthesized dsRNA were assessed by Nanodrop2000 (Thermo Fisher Scientific, Waltham, MA, USA) and electrophoresis on 1% agarose gel respectively. All the purified dsRNA was stored at −80 °C for further experiment.

In order to optimize the silencing efficiency of *LvHSSP* dsRNA, shrimp were injected with different dosages of *LvHSSP* dsRNA (0.5 µg/g, 1 µg/g, and 2 µg/g) and an equal amount of EGFP dsRNA was used as the control. At 48 h post-dsRNA injection, the transcription level of *LvHSSP* in hemocytes of at least four individuals was detected and the dosage of 1 µg/g was selected for further RNAi experiments.

In the RNAi experiment, shrimp were randomly divided into two groups, including dsEGFP and dsLvHSSP groups. DsRNA of EGFP and LvHSSP genes were injected into the last abdominal segment of each shrimp at the final concentration of 1 µg/g, respectively. After 6, 12, and 24 h post-dsRNA injection, five individuals were used for total hemocyte counting in each group. At 48 h post-dsRNA injection, each shrimp in different groups was challenged with 2000 copies of WSSV. Hemocytes from 15 individuals (five individuals in one biological replicate) in each group were collected at 24 hpi and 48 hpi and immediately preserved in liquid nitrogen for RNA extraction. The pleopods of 15 individuals (three individuals in one biological replicate) in each group were collected at 24 hpi and 48 hpi and preserved in liquid nitrogen for DNA extraction to detect the virus load.

### 2.5. Recombinant Expression and Purification of LvHSSP

The nucleotide sequence encoding mature LvHSSP was amplified by primers LvHSSP-pMAL-His-F and LvHSSP-pMAL-His-R (Table S1) and inserted into the pMAL-c5x vector (TIANZA, Beijing, China) using the In-Fusion^®^ HD Cloning Kit (Clontech, Mountain View, CA, USA) to construct pMAL-LvHSSP-His recombinant expression plasmid. The constructed recombinant plasmid was then transformed into *E. coli* TransB (DE3) chemically competent cells (TransGen Biotech, Beijing, China) and the sufficient fusion protein was induced with isopropyl-beta-D-thiogalactopyranoside (IPTG, Solarbio, Beijing, China) at a final concentration of 0.2 mM at 16 °C for 24 h. The cells were collected and sonicated to release the intracellular fusion protein. After centrifugation at 12,000× *g* for 10 min at 4 °C, target protein (designated as rMBP-HSSP-His) was purified by TALON^®^ Metal Affinity Resin (Takara, Kyoto, Japan) following the manufacturer’s instructions. The purified protein was qualified by SDS-PAGE and its concentration was detected using a BCA protein quantification kit (Vazyme Biotech, Nanjing, China). Maltose binding protein (MBP) with His-tag was concurrently induced and purified as a negative control (designated as rMBP-His).

In order to further confirm the effect of LvHSSP during WSSV infection, shrimp were divided into two groups (30 individuals in each group). The purified rLvHSSP protein was mixed with WSSV and immediately intramuscularly injected into shrimp (10 μg/shrimp). The MBP-His tag mixed with WSSV was injected as the negative control. The pleopods of 15 individuals (three individuals in one biological replicate) in each group were collected at 24 hpi and 48 hpi and preserved in liquid nitrogen for DNA extraction to detect the virus load.

### 2.6. DNA Extraction and WSSV Load Quantification

DNA was extracted from pleopods using the Genomic DNA Kit (Tiangen, Beijing, China) according to the manufacturer’s instructions. Protease K (Roche, Mannheim, Germany) was added at a final concentration of 5.7 mg/mL for digestion. Extracted DNA was quantified by NanoDrop 2000 (Thermo Fisher Scientific, Waltham, MA, USA). Viral loads in the pleopods were quantitatively analyzed using SYBR Green-based quantitative real-time PCR (qPCR) according to the method described by Sun et al. [24]. Briefly, The DNA encoding the extra-cellular part of the WSSV envelope protein VP28 was amplified and cloned into the pMD19-T simple vector (TaKaRa, Kyoto, Japan). The purified and quantified plasmid was used to generate a standard curve. The DNA of the pleopods was used to detect the viral loads with primers VP28-qF and VP28-qR (Table S1). Each assay was carried out in quadruplicate.

### 2.7. Total Hemocytes Counting

The total number of hemocytes in shrimps was detected as previously described [27]. Briefly, 100 μL hemolymph was extracted from the sinus of each shrimp from different groups (dsEGFP group, dsHSSP group, dsHSSP + rMBP-His group, and dsHSSP + rMBP-HSSP-His group), and mixed with the same amount of sterile anticoagulant after 6, 12, and 24 h post-dsRNA injection. Trypan blue was added in a ratio of 1/5 to detect dead cells. The number of hemocytes in each shrimp was counted five times by hemocytometer under the optical microscope (Nikon ECLIPSE 80i, Nikon, Tokyo, Japan).

### 2.8. Immunocytochemical Assay

The immunocytochemical assay was conducted as previously described [28] with some modification. Briefly, hemocytes collected from healthy shrimp were suspended in the 5% BSA containing resuspending solution [29], seeded in a cell culture dish (NEST, Wuxi, China) for 3 h, and incubated with rMBP-HSSP-His (50 μg/mL) at room temperature. The same concentration of rMBP-His was used as the negative control. The hemocytes were fixed on dishes with 4% paraformaldehyde prepared in PBS (pH 7.4) for 15 min at room temperature, and washed three times in TBS (50 mM, Tris–HCl, 150 mM NaCl, pH 7.4). After blocking in 5% BSA in TBS at 4 °C overnight, the hemocytes were incubated with mouse anti-MBP antibody (ABclonal, Wuhan, China) in a dilution of 1:500 at room temperature for 1 h, and then incubated with Alexa Fluor 488-conjugated goat anti-mouse secondary antibody (Abcam, Shanghai, China) at a dilution of 1:1000 at room temperature for 1 h. Hemocytes were dyed with Di1 (Beyotime, Shanghai, China) for 20 min and DAPI (Beyotime, Shanghai, China) for 5 min before the observation under a laser confocal scanning microscopy (Carl Zeiss LSM 710, Carl Zeiss, Oberkochen, Germany).

### 2.9. Cell Culture, Transfection, Co-Immunoprecipitation and Western Blot Assay

Green fluorescent protein (GFP) signal observation after site-mutant and co-immunoprecipitation (Co-IP) assays were performed in Sf9 cells to detect the code capacity of LvHSSP and its interaction with WSSV envelope proteins. Sf9 cells were purchased from the China Center for Type Culture Collection (Wuhan, China), cultured in Sf-900™ II SFM (Gibco, Grand Island, NE, USA) at 27 °C, and sub-cultured every 3–4 days. The nucleotide sequence encoding the mature peptide of LvHSSP and ORFs encoding VP19, VP24, VP26, and VP28 of WSSV were amplified by primers listed in Table S1 and inserted into pDHsp70-Flag/His and pDHsp70-V5/His vectors (generously provided by Pro Lo [30]) for Flag or V5 fusion protein expression. The mutation of signal peptide and full length of wild-type ORF of LvHSSP were amplified by primers listed in Table S1 [31] and inserted into the pDHsp70-GFP-Flag/His vector for GFP fusion protein expression. The primers D70GFPmut-F and D70GFPmut-R were used to construct the site-mutant plasmid of GFP (GFPmut), in which the start code “ATGGTG” of pDHsp70-GFP-Flag/His (GFPwt) vector was mutated into “ATTGTT”. The ORF, 5′-UTR-ORF, and 5′UTR-ORFmut of *LvHSSP* were amplified by primers listed in Table S1 and inserted into the GFPmut vector to construct the ORF-GFPmut, 5′-UTR-ORF-GFPmut, and 5′UTR-ORFmut-GFPmut expression plasmids, respectively [32]. Different plasmids (pDHsp-Flag-HSSP^WSP^, pDHsp-V5-VP19, pDHsp-V5-VP24, pDHsp-V5-VP26, pDHsp-V5-VP28, pDHsp-V5, pDHsp-Flag, GFPwt, GFPmut, ORF-GFPmut, 5′-UTR-ORF-GFPmut, and 5′UTR-ORFmut-GFPmut) were transfected into Sf9 cells using Lipofectamine 3000 reagent (Life Technologies, Carlsbad, CA, USA) according to the manufacturer’s instructions. After being cultured at 27 °C overnight, the cells were subjected to heat-shock treatment (42 °C for 30 min) and subsequently cooled to 27 °C.

For the code capacity assay, the Sf9 cells transfected with different plasmids were observed at 24 h after the heat shock treatment under the green fluorescence signal and bright field channels using a Nikon Eclipse Ti-S microscope (Nikon, Tokyo, Japan). For the Co-IP assay, the cells were harvested in cell lysis buffer (Beyotime, Shanghai, China) at 24 h after the heat shock treatment. Input samples were prepared from the cell lysate, and the remaining lysates were mixed with anti-Flag M2 magnetic beads (Sigma, San Francisco, CA, USA) under gentle shaking on a roller at 4 °C for 2 h. The beads were then washed two times with PBS and cell lysis buffer. Input and Co-IP samples were incubated with 4× LDS sample buffer (GenScript, Nanjing, China) at 100 °C for 10 min. Protein was analyzed by Western blotting using anti-V5 and anti-Flag antibodies (Cell Signaling Technology, Danvers, MA, USA).

For the secretory ability assay, the cells in a 6-well plate and culture medium were collected at 24 h after the heat shock treatment. The cells were lysed at 4 °C for 10 min by cell lysis buffer (Beyotime, Shanghai, China). The protein in medium was collected with saturated ammonium sulfate and resuspended with PBS buffer. The samples were incubated with 4× LDS sample buffer (GenScript, Nanjing, China) at 100 °C for 10 min and detected by Western blotting using anti-GFP and anti-GADPH antibodies (ABclonal, Wuhan, China). All experiments were performed in triplicates.

### 2.10. Statistical Analysis

All data were presented in the form of the mean ± S.E except for the THC. The THC was presented as the results of each individual and their median values were marked with a line. An independent sample *t*-test was applied to analyze whether the difference between the two groups was significant by SPSS 19.0 software (SPSS, Chicago, IL, USA). The significant differences for *p* < 0.05 and *p* < 0.01 were represented by one star and two stars, respectively.

## 3. Results

### 3.1. LvHSSP Is a Hemocyte-Specific Peptide and Responsive to WSSV Infection

**Figure 1:**
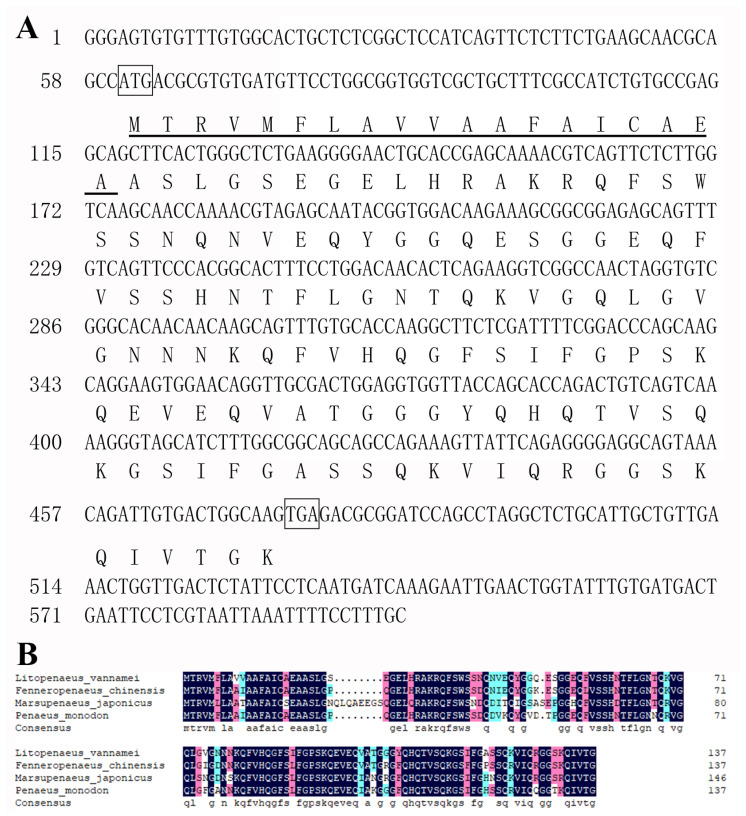
Sequence of LvHSSP. (**A**) Diagram of structure of LvHSSP transcript. (**B**) Alignment of amino acid sequences of LvHSSP and its homologs in other three species of shrimp. GenBank accession numbers of sequences from *L. vannamei*, *F. chinensis*, *M. japonicus*, and *Penaeus monodon*, were XM_027350838, XM_047642709, LC349927, and XM_037932926, respectively.

The transcript of LvHSSP obtained from the transcriptome database of *L. vannamei* was validated by Sanger sequencing. The full length of this transcript was 599 base pairs (bp), containing a predicted ORF with 417 bp and 5′-UTR with 60 bp (Figure 1A). The predicted ORF encoded 138 amino acids (aa), including a 19 aa signal peptide and the following peptide without functional annotation. From the NCBI database, nucleotide sequences encoding similar peptides could be found in other shrimp species (Figure 1B), but not in other animals.

**Figure 2:**
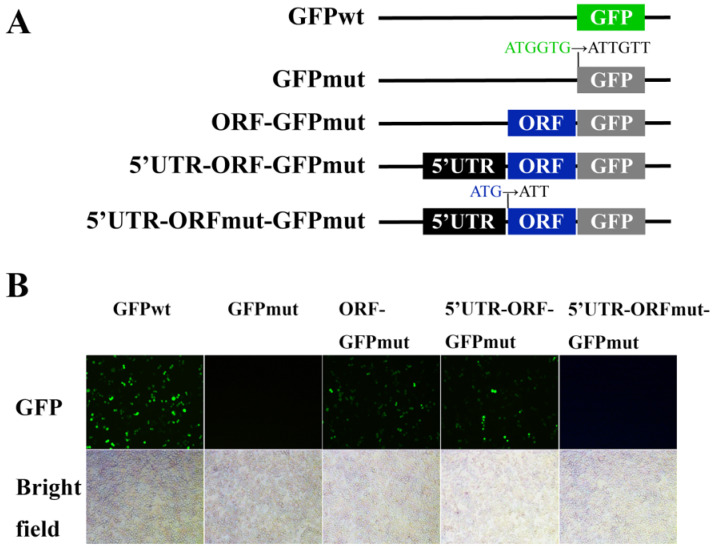
Encoding capability of start codon in the predicted ORF in LvHSSP transcript. (**A**) Diagram of the GFP fusion constructs used for transfection. The start codon ATGGTG of the GFP (GFPwt) gene is mutated to ATTGTT (GFPmut). The start codon ATG of the ORF in LvHSSP transcript is mutated to ATT. (**B**) GFP fluorescence signals in different constructs of transfected Sf9 cells.

To investigate whether the start codon of the ORF in LvHSSP was active, a series of plasmid constructs were generated in which a GFPmut ORF (the start codon ATGGTG is mutated to ATTGTT) was fused to the C terminus of the ORF and the 5′UTR-ORF of the LvHSSP transcript (Figure 2A). Substantial expression of the LvHSSP-GFP fusion protein was observed in LvHSSP ORF-GFPmut- and LvHSSP 5′UTR-ORF-GFPmut-transfected Sf9 cells (Figure 2B). However, mutation of the start codon in the ORF of *LvHSSP* transcript from ATG to ATT (5′UTR-ORFmut-GFPmut) failed to express the LvHSSP-GFP fusion protein (Figure 2B). As controls, green fluorescence signal could be observed in the GFPwt-transfected Sf9 cells, but none in the GFPmut-transfected cells (Figure 2B).

**Figure 3:**
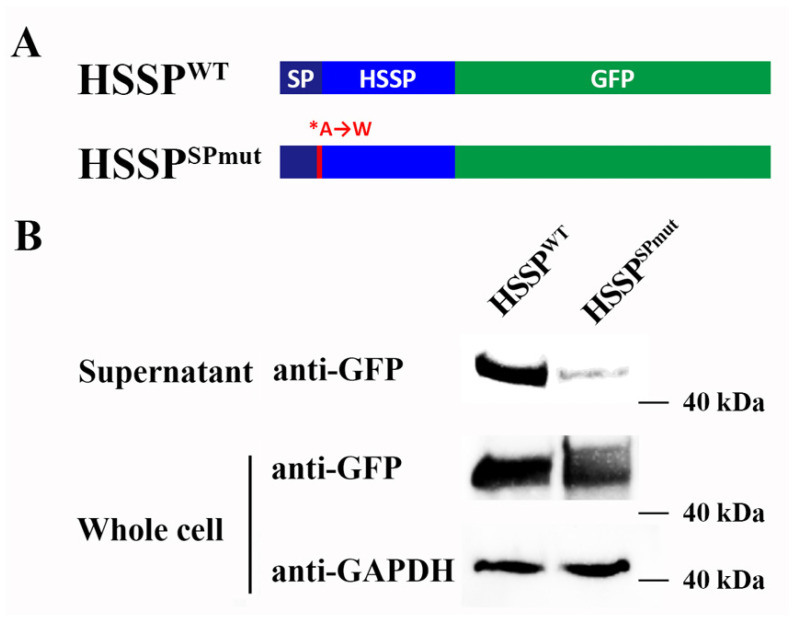
LvHSSP signal peptide sequence drove secretion. (**A**) Diagram of the GFP fusion constructs used for transfection. The 19th A residue in the signal peptide of LvHSSP (LvHSSP^WT^) is mutated to W (LvHSSP^SPmut^). (**B**) Western blotting results showed that transfection with plasmids encoding LvHSSP-GFP fusion proteins revealed that the wild-type LvHSSP signal peptide sequence (HSSP^WT^) drove secretion (band of GFP in the supernatant), whereas mutation of A → W in the signal peptide cleavage site (HSSP^SPmut^) caused the reduction of signals in the supernatant.

To investigate whether LvHSSP encoded a secretory protein, the mutant nucleotide sequence encoding the signal peptide and wild-type ORF of LvHSSP were fused to the N terminus of GFP, respectively. The bands of LvHSSP-GFP fusion protein were observed in the serum and whole cell lysis of LvHSSP^WT^-GFP-transfected Sf9 cells. However, the bands could be observed in the whole cell lysis of LvHSSP^SPmut^-GFP-transfected Sf9 cells, but nearly undetectable in the corresponding serum compared to that in the serum of LvHSSP^WT^-GFP-transfected Sf9 cells (Figure 3).

**Figure 4:**
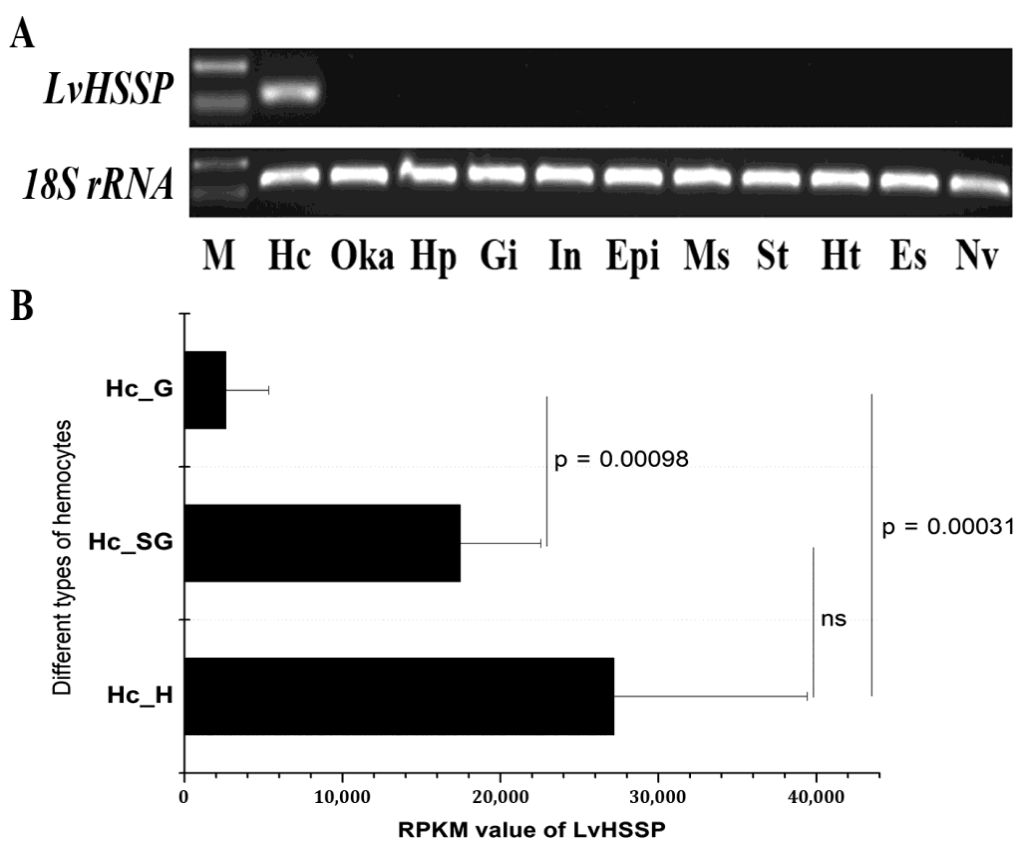
Distribution of *LvHSSP* among different tissues and different types of hemocytes. (**A**) Expression patterns of *LvHSSP* in different tissues of *L. vannamei*. The *18S rRNA* gene was used as the internal reference. Hc, hemocytes; Oka, lymphoid organ; Hp, hepatopancreas; Gi, gill; In, intestine; Epi, epidermis; Ms, muscle; St, stomach; Ht, heart. (**B**) RPKM values of *LvHSSP* in different types of hemocytes. Hc_H, hyalinocytes; Hc_SG, semigranulocytes; Hc_G, granulocytes. The *p*-values among three subpopulations were listed.

**Figure 5:**
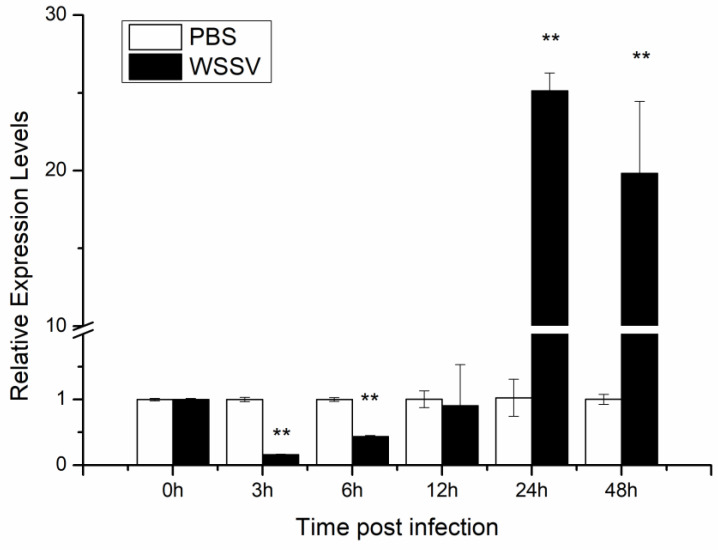
Expression levels of *LvHSSP* in hemocytes of shrimp at different time post-WSSV challenge. PBS stands for PBS injection group, and WSSV stands for WSSV injection group. Stars (**) indicate extremely significant difference (*p* < 0.01) in the gene expression levels between the two treatments. All assays described above were biologically repeated three times.

Tissue distribution of *LvHSSP* in shrimp was analyzed by RT-PCR, and the results showed that the transcripts of *LvHSSP* were specifically expressed in hemocytes (Figure 4A). Transcriptome data showed that *LvHSSP* was highly expressed in hyalinocytes and semi-granulocytes (Figure 4B). To determine whether *LvHSSP* is responsive to WSSV infection, the expression pattern of *LvHSSP* in the hemocytes of shrimp during WSSV infection was analyzed by qPCR. The expression of *LvHSSP* significantly decreased in hemocytes at 3 hpi and 6 hpi, but significantly increased at 24 hpi and 48 hpi (Figure 5). These results suggested that LvHSSP was a hemocyte-specific secretory protein and might be involved in viral infection in shrimp.

### 3.2. LvHSSP Facilitates WSSV Proliferation in Shrimp

**Figure 6:**
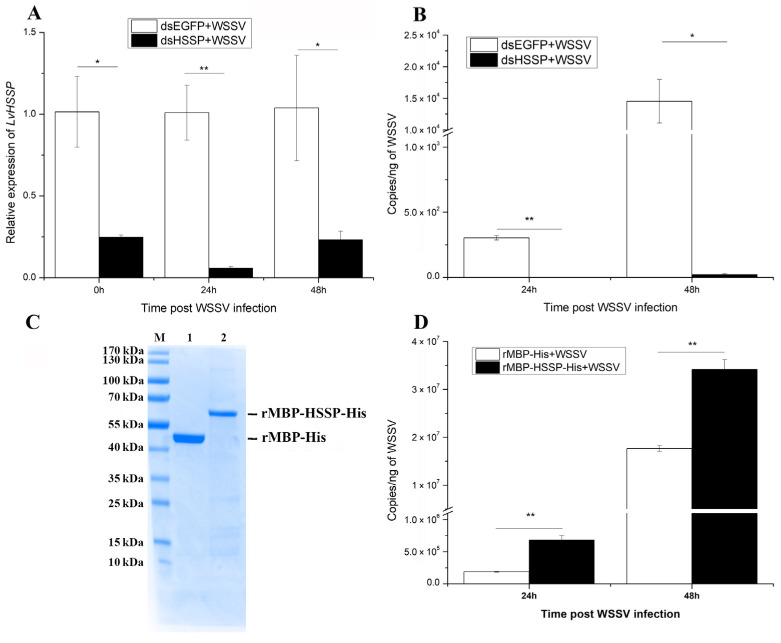
LvHSSP is beneficial for WSSV infection in shrimp. (**A**) Inhibition efficiency of *LvHSSP* dsRNA. Expression levels of *LvHSSP* in hemocytes of dsHSSP + WSSV and control groups after 0, 24, and 48 h post-WSSV infection. (**B**) Amount of WSSV particles in different groups at different hours after silencing of *LvHSSP* and WSSV infection. (**C**) Expression and purification of recombinant of rMBP-HSSP-His. Lane M, protein marker; lane 1, purified control protein rMBP-His; lane 2, purified recombinant protein rMBP-HSSP-His. (**D**) Amount of WSSV particles in different groups at different hours after infection of the incubated mixture of recombinant protein and WSSV. Stars (*) indicate significant difference (*p* < 0.05) and (**) indicate extremely significant difference (*p* < 0.01) in the gene expression levels between the two treatments. All assays described above were biologically repeated at least three times.

To characterize the function of LvHSSP during WSSV infection, RNAi and subsequential WSSV infection were conducted. After dosage optimization, 1 μg dsRNA per gram of body weight led to a down-regulation of *LvHSSP* by 75.2% at 48 h post-dsRNA injection, and kept 75~95% knockdown efficiency during the subsequential WSSV infection (Figure 6A). The WSSV copy numbers in pleopods of shrimp from dsEGFP + WSSV and dsHSSP + WSSV groups were detected at 24 hpi and 48 hpi to assess the viral propagation after *LvHSSP* knockdown. The viral copy numbers in dsEGFP + WSSV group were about 3.05 × 10^2^ and 1.45 × 10^4^ copies ng^−1^ DNA at 24 hpi and 48 hpi, while they were much lower (*p* < 0.01) in shrimp from the dsHSSP + WSSV group, about 1.48 and 22.2 copies ng-1 DNA at 24 hpi and 48 hpi, respectively (Figure 6B).

To further confirm the function of LvHSSP in the antiviral immunity of shrimp, an overexpression assay using recombinant protein was performed. The MBP-HSSP-His fusion protein and MBP-His control protein were expressed and purified (Figure 6C) and incubated with WSSV at room temperature for 2 h. After the mixture was injected into the shrimp for 24 h and 48 h, the viral loads of WSSV were analyzed. The viral copies in rMBP-His + WSSV group were about 1.87 × 10^5^ and 1.77 × 10^7^ copies ng^−1^ DNA at 24 hpi and at 48 hpi, while they were much higher (*p* < 0.01) in shrimp from the rMBP-HSSP-His + WSSV group, about 6.81 × 10^5^ and 3.41 × 10^7^ copies ng-1 DNA at 24 hpi and 48 hpi, respectively (Figure 6D). These data suggested that LvHSSP facilitates WSSV propagation in shrimp.

### 3.3. LvHSSP Interacts with VP26 of WSSV and Binds to Shrimp Hemocytes

**Figure 7:**
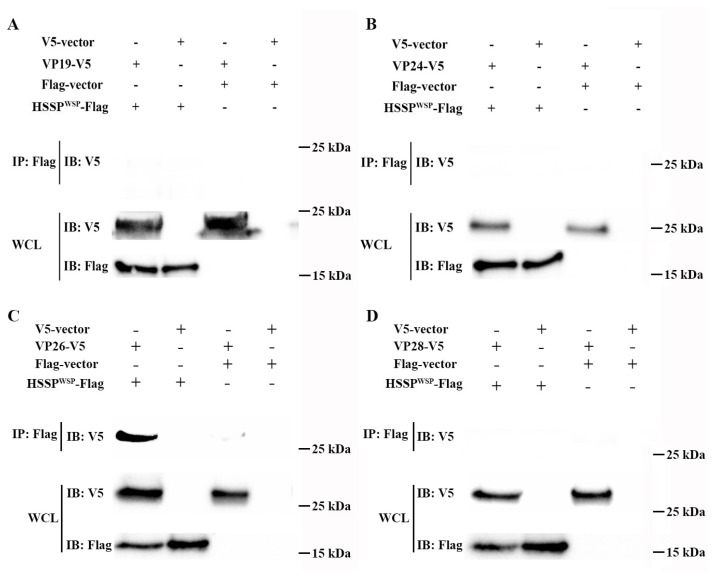
Interaction between LvHSSP and WSSV envelope protein. (**A**) Determination of the interaction between LvHSSP and VP19 by a Co-IP assay. Co-IP results showed that LvHSSP cannot interact with VP19. (**B**) Determination of the interaction between LvHSSP and VP24 by a Co-IP assay. Co-IP results showed that LvHSSP cannot interact with VP24. (**C**) Determination of the interaction between LvHSSP and VP26 by a Co-IP assay. Co-IP results showed that LvHSSP directly interacts with VP26. (**D**) Determination of the interaction between LvHSSP and VP28 by a Co-IP assay. Co-IP results showed that LvHSSP cannot interact with VP28. Anti-V5 Western blot bands show the expression of VP19-V5, VP24-V5, VP26-V5, or VP28-V5, and anti-Flag Western blot bands show the expression of LvHSSP-Flag.

To study the possible mechanism of LvHSSP activity during WSSV infection, its interaction with the four main envelope proteins of WSSV was analyzed using a Co-IP assay. The results showed that LvHSSP could interact with VP26 (Figure 7C), but not with VP19 (Figure 7A), VP24 (Figure 7B), or VP28 (Figure 7D). These results suggested LvHSSP probably participated in WSSV infection via interaction with VP26.

**Figure 8:**
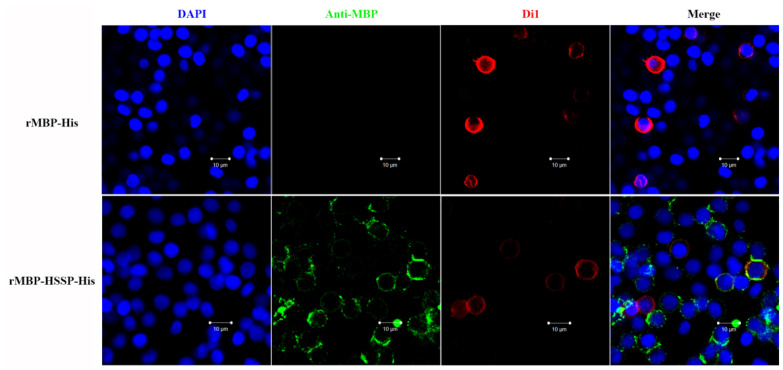
Co-location of LvHSSP protein and the hemocytes. rMBP-His represents the results of MBP tag protein, and rMBP-HSSP-His represents the results of LvHSSP recombinant protein. The blue signal of DAPI indicates the nucleus, the green signal of anti-MBP indicates the positive signals of target proteins, the red signal of Di1 indicates the membrane, and the last lanes are the merged picture of previous three signals. Bar = 10 μm.

As LvHSSP could interact with VP26, it might mediate WSSV infection in hemocytes. To test this hypothesis, recombinant LvHSSP protein (rMBP-HSSP-His) was incubated with shrimp hemocytes in vitro. After washing, the existence of recombinant LvHSSP protein on the surface of hemocytes was detected by immunofluorescence. Under laser scanning confocal microscopy, the positive signal of Alexa Fluor^®^ 488 conjugated antibody was indicated by green fluorescence, while the membrane and nucleus of shrimp hemocytes were in red and blue fluorescence, respectively. There was no positive signal in the rMBP-His control, and obviously positive signals representing recombinant LvHSSP protein could be detected on the surface of shrimp hemocytes (Figure 8). These results indicated that WSSV probably targeted LvHSSP to achieve its infection in shrimp hemocytes.

### 3.4. LvHSSP Modulated the Number of Total Circulating Hemocytes

**Figure 9:**
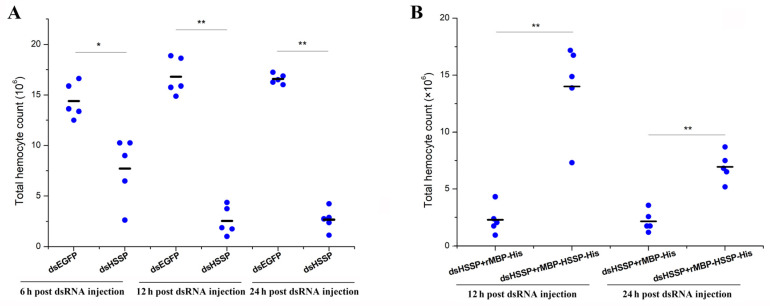
The effect of LvHSSP on the total hemocyte counts. (**A**) The total hemocyte counts after knockdown of *LvHSSP*. dsEGFP, injected with EGFP dsRNA; dsHSSP, injected with *LvHSSP* dsRNA. (**B**) The total hemocyte counts after knockdown of *LvHSSP* and rescue with recombinant LvHSSP protein. dsHSSP + rMBP-His, injected with *LvHSSP* dsRNA and rMBP-His tag protein; dsHSSP + rMBP-HSSP-His, injected with *LvHSSP* dsRNA and recombinant rMBP-HSSP-His protein. Bar, median of THC. Stars (*) indicate significant difference (*p* < 0.05) and (**) indicate extremely significant difference (*p* < 0.01) in the THC between the two treatments.

Notably, pre-experiment results showed that less hemocytes could be collected after knockdown of *LvHSSP*. Therefore, we counted the total hemocytes number after different treatments. The total hemocyte counts (THC) with intact morphology in the dsEGFP control group were about 1.44 × 10^7^ cell/mL, 1.68 × 10^7^ cell/mL, and 1.68 × 10^7^ cell/mL at 6, 12, and 24 h post-dsRNA injection, respectively. After *LvHSSP* knockdown, the THC in shrimp were about 7.73 × 10^6^ cell/mL, 2.55 × 10^6^ cell/mL, and 2.68 × 10^6^ cell/mL at 6, 12, and 24 h post-dsRNA injection, respectively, which was significantly less than those in the dsEGFP control group (Figure 9A). In the rescue experiment, the THC in shrimp after *LvHSSP* knockdown and subsequent rMBP-HSSP-His injection (dsHSSP + rMBP-HSSP-His) were about 1.40 × 10^7^ cell/mL and 6.94 × 10^6^ cell/mL at 12 and 24 h post-treatment, which was much more than those in shrimp after *LvHSSP* knockdown and subsequent MBP-tag protein injection (dsHSSP + rMBP-His), about 2.29 × 10^6^ cell/mL and 2.16 × 10^6^ cell/mL at 12 and 24 h post-treatment, respectively (Figure 9B). The results suggested that LvHSSP might facilitate WSSV infection by modulating the number of circulating hemocytes in shrimp.

## 4. Discussion

Nowadays, numerous previously unannotated transcripts have become a fabulous source for exploring novel proteins, which might exhibit diverse functions in multiple biological processes. The genome sequence of *L. vannamei* covered 25,596 protein-coding genes and a large scale of unannotated transcripts [22]. Like the human genome, these unannotated transcripts contain numerous ncRNAs and uncharacterized RNAs, and some of them might be capable of encoding novel peptides, which participate in multiple physiological processes [33]. In the present study, we identified a transcript (LvHSSP) specifically expressed in hemocytes without any annotation previously. Mutant of the signal peptide led to a remarkable reduction in the LvHSSP fusion protein in the serum, indicating LvHSSP was a secretory protein. Actually, the band of the fusion protein in the serum should be undetectable in the mutant group, but a weak corresponding band was observed in the present study, and it might result from the disruption of several cells and outflow of cell contents after high-temperature treatment, which promotes the expression of the fusion protein from the HSP70 promoter containing plasmid [30]. From the transcriptome data of hemocytes, we found that its expression level ranked top 5 in hemocytes based on the RPKM value [22]. In addition, *LvHSSP* displayed an obvious response to WSSV infection. Therefore, its role in hemocyte immunity should be an interesting issue for further investigation.

Previous studies reported that some shrimp proteins could facilitate the infection process of WSSV by binding the main envelope proteins, including VP19, VP24, VP26, and VP28, which are vital for virus infection [34]. Some cuticle proteins from *L. vannamei*, LvAMP13.4 and LvCPAP1, could favor the entry process of WSSV by binding VP24 [35,36]. LvCPG2, the chondroitin sulfate proteoglycans from *L. vannamei*, facilitated WSSV infection by interacting with VP26 and VP28 [37]. LvHSSP could also interact directly with the envelope protein, VP26, of WSSV. Among the main envelope proteins, VP28 and VP26 are the most abundant, accounting for approximately 60% of the envelope [38,39]. VP26 acts as a linker protein between the envelope and the nucleocapsid [38]. It is capable of binding to actin or actin-associated proteins to facilitate virus entry [40,41]. Together with the result of LvHSSP directly binding to the hemocyte membrane surface, the interaction between LvHSSP and VP26 suggested that WSSV might target this protein for its adhesion and entry into host cells.

Cell adhesion is essential for many physiological processes and pathological conditions [42]. Selectins are one kind of mammalian cell adhesion molecule, and they are important for the regulation of lymphocyte recirculation during the inflammatory response [43]. The first purified and well-studied invertebrate adhesion protein is crayfish peroxinectin. It is synthesized in hemocytes and mediates hemocytes attachment and spreading in response to stimulus [44]. The freely circulating hemocytes in *Drosophila* larvae can adhere to the body wall after wounding or cluster at sites of infection [45]. Knockdown of LvHSSP led to a decrease of THC and could be rescued by supplementing with LvHSSP protein, suggesting that LvHSSP might modulate the spread of hemocytes. Based on the present data, we speculate that although the immune function of LvHSSP remained to be elucidated, the virus probably targeted this immune and hemocyte-associated protein to achieve infection on hemocytes and the delivery of virus in shrimp.

Hemocytes play central roles in the shrimp immune system. They are mainly classified into granulocytes, semigranulocytes, and hyalinocytes depending on the morphology and intracellular granule staining characteristics [46]. These three subpopulations exhibited different functions with significantly distinct gene expression patterns. Our previous study showed that phagocytosis-related genes were highly expressed in hyalinocytes, and together with the functional analysis results, suggested that hyalinocytes might be the main type of hemocytes that possess the capability to ingest foreign materials. Besides the hyalinocytes, semi-granulocytes highly expressed genes involved in receptor-dependent endocytosis, which could also engulf foreign particles [25]. Hemocytes are also the target cells for WSSV infection [47]. In the present study, we found that LvHSSP was mainly expressed in hyalinocytes and semi-granulocytes. Therefore, hyalinocytes and semi-granulocytes might be the target cells for LvHSSP binding and WSSV infection. Further molecular mechanisms such as the identification of the putative LvHSSP receptor on the membrane of hemocytes still need to be investigated.

## Data Availability

Not applicable.

## Supplementary Materials

The following supporting information can be downloaded at: https://www.mdpi.com/article/10.3390/v15010227/s1. Table S1: Primer sequences used in the functional study of *LvHSSP* genes.

## References

[1] Li F.H., Xiang J.H. (2013). Recent advances in researches on the innate immunity of shrimp in China. Dev. Comp. Immunol..

[2] Li F., Xiang J. (2013). Signaling pathways regulating innate immune responses in shrimp. Fish Shellfish Immun..

[3] Zhang X., Song X., Huang J. (2016). Impact of Vibrio parahaemolyticus and white spot syndrome virus (WSSV) co-infection on survival of penaeid shrimp Litopenaeus vannamei. Chin. J. Oceanol. Limn..

[4] Chou H.-Y., Cy H., Ch W., Hc C., Lo C. (1995). Pathogenicity of a baculovirus infection causing white spot syndrome in cultured penaeid shrimp in Taiwan. Dis. Aquat. Organ..

[5] Li C.Z., Wang S., He J.G. (2019). The Two NF-kappa B Pathways Regulating Bacterial and WSSV Infection of Shrimp. Front. Immunol..

[6] Watson A., Agius J., Ackerly D., Beddoe T., Helbig K. (2022). The Role of Anti-Viral Effector Molecules in Mollusc Hemolymph. Biomolecules.

[7] Zhao X.F., Wang J.X. (2008). The antimicrobial peptides of the immune response of shrimp. ISI-Invert. Surviv. J..

[8] Li S., Li F. (2020). The Anti-lipopolysaccharide Factors in Crustaceans. Subcell Biochem.

[9] Rosa R.D., Barracco M.A. (2010). Antimicrobial peptides in crustaceans. ISI-Invert. Surviv. J..

[10] Maningas M.B., Kondo H., Hirono I., Saito-Taki T., Aoki T. (2008). Essential function of transglutaminase and clotting protein in shrimp immunity. Mol. Immunol..

[11] Maningas M.B.B., Kondo H., Hirono I. (2013). Molecular mechanisms of the shrimp clotting system. Fish Shellfish Immunol..

[12] Soetrisno C.K. (2009). Non-clotting haemolymph of WSSV-infected shrimp: Is it a factor in infection processes?. Indones. Aquac. J..

[13] Sahul Hameed A.S., Sarathi M., Sudhakaran R., Balasubramanian G., Syed Musthaq S. (2006). Quantitative assessment of apoptotic hemocytes in white spot syndrome virus (WSSV)-infected penaeid shrimp, *Penaeus monodon* and *Penaeus indicus*, by flow cytometric analysis. Aquaculture.

[14] Sellos D., Lemoine S., Van Wormhoudt A. (1997). Molecular cloning of hemocyanin cDNA from *Penaeus vannamei* (Crustacea, Decapoda): Structure, evolution and physiological aspects 1EBI sequence accession number: X82502.1. FEBS Lett..

[15] Zhang X., Huang C., Qin Q. (2004). Antiviral properties of hemocyanin isolated from shrimp *Penaeus monodon*. Antivir. Res..

[16] Lei K., Li F., Zhang M., Yang H., Luo T., Xu X. (2008). Difference between hemocyanin subunits from shrimp *Penaeus japonicus* in anti-WSSV defense. Dev. Comp. Immunol..

[17] Zhan S., Aweya J.J., Wang F., Yao D., Zhong M., Chen J., Li S., Zhang Y. (2019). Litopenaeus vannamei attenuates white spot syndrome virus replication by specific antiviral peptides generated from hemocyanin. Dev. Comp. Immunol..

[18] Liu S., Aweya J.J., Zheng L., Wang F., Zheng Z., Zhong M., Lun J., Zhang Y. (2018). A Litopenaeus vannamei Hemocyanin-Derived Antimicrobial Peptide (Peptide B11) Attenuates Cancer Cells’ Proliferation. Molecules.

[19] Liu S., Aweya J.J., Zheng L., Zheng Z., Huang H., Wang F., Yao D., Ou T., Zhang Y. (2022). LvHemB1, a novel cationic antimicrobial peptide derived from the hemocyanin of *Litopenaeus vannamei*, induces cancer cell death by targeting mitochondrial voltage-dependent anion channel 1. Cell Biol. Toxicol..

[20] Zhao B.-R., Zheng Y., Gao J., Wang X.-W. (2020). Maturation of an Antimicrobial Peptide Inhibits *Aeromonas hydrophila* Infection in Crayfish. J. Immunol..

[21] Diao M.-Q., Li C., Xu J.-D., Zhao X.-F., Wang J.-X. (2019). RPS27, a sORF-Encoded Polypeptide, Functions Antivirally by Activating the NF-κB Pathway and Interacting With Viral Envelope Proteins in Shrimp. Front. Immunol..

[22] Zhang X., Yuan J., Sun Y., Li S., Gao Y., Yu Y., Liu C., Wang Q., Lv X., Zhang X. (2019). Penaeid shrimp genome provides insights into benthic adaptation and frequent molting. Nat. Commun..

[23] Rodriguez J., Boulo V., Mialhe E., Bachere E. (1995). Characterisation of shrimp haemocytes and plasma components by monoclonal antibodies. J. Cell Sci..

[24] Sun Y., Li F., Xiang J. (2013). Analysis on the dynamic changes of the amount of WSSV in Chinese shrimp *Fenneropenaeus chinensis* during infection. Aquaculture.

[25] Sun M., Li S., Zhang X., Xiang J., Li F. (2020). Isolation and transcriptome analysis of three subpopulations of shrimp hemocytes reveals the underlying mechanism of their immune functions. Dev. Comp. Immunol..

[26] Wang Z.W., Li S.H., Li F.H., Xie S.J., Xiang J.H. (2016). Identification and function analysis of a novel vascular endothelial growth factor, LvVEGF3, in the Pacific whiteleg shrimp Litopenaeus vannamei. Dev. Comp. Immunol..

[27] Li X., Li S., Sun M., Yu Y., Zhang X., Xiang J., Li F. (2022). A newly identified NLR-like gene participates in bacteria and virus infection possibly through regulating hemocytes apoptosis in shrimp. Dev. Comp. Immunol..

[28] Xin L., Zhang H., Zhang R., Li H., Wang W., Wang L., Wang H., Qiu L., Song L. (2015). CgIL17-5, an ancient inflammatory cytokine in *Crassostrea gigas* exhibiting the heterogeneity functions compared with vertebrate interleukin17 molecules. Dev. Comp. Immunol..

[29] Sun M.Z., Wang L.L., Jiang S., Liu R., Zhao D.P., Chen H., Song X.R., Song L.S. (2015). CpG ODNs induced autophagy via reactive oxygen species (ROS) in Chinese mitten crab, Eriocheir sinensis. Dev. Comp. Immunol..

[30] Chang Y.-S., Liu W.-J., Lee C.-C., Chou T.-L., Lee Y.-T., Wu T.-S., Huang J.-Y., Huang W.-T., Lee T.-L., Kou G.-H. (2010). A 3D model of the membrane protein complex formed by the white spot syndrome virus structural proteins. PLoS ONE.

[31] Pauli A., Norris M.L., Valen E., Chew G.-L., Gagnon J.A., Zimmerman S., Mitchell A., Ma J., Dubrulle J., Reyon D. (2014). Toddler: An Embryonic Signal That Promotes Cell Movement via Apelin Receptors. Science.

[32] Huang J.-Z., Chen M., Chen D., Gao X.-C., Zhu S., Huang H., Hu M., Zhu H., Yan G.-R. (2017). A Peptide Encoded by a Putative lncRNA HOXB-AS3 Suppresses Colon Cancer Growth. Mol. Cell.

[33] Xing J., Liu H., Jiang W., Wang L. (2020). LncRNA-Encoded Peptide: Functions and Predicting Methods. Front. Oncol..

[34] Van Etten J. (2009). Lesser known large dsDNA viruses. Preface. Curr. Top. Microbiol. Immunol..

[35] Yang F., Li S., Li F., Xiang J. (2018). A cuticle protein from the Pacific white shrimp *Litopenaeus vannamei* involved in WSSV infection. Dev. Comp. Immunol..

[36] Yang F., Li X., Li S., Xiang J., Li F. (2020). A novel cuticle protein involved in WSSV infection to the Pacific white shrimp *Litopenaeus vannamei*. Dev. Comp. Immunol..

[37] Ren X.-C., Liu Q.-H. (2021). LvCPG2 facilitated WSSV infection by interaction with VP26 and VP28. Fish Shellfish Immun..

[38] Sánchez-Paz A. (2010). White spot syndrome virus: An overview on an emergent concern. Vet. Res..

[39] Tang X., Wu J., Sivaraman J., Hew C.L. (2007). Crystal structures of major envelope proteins VP26 and VP28 from white spot syndrome virus shed light on their evolutionary relationship. J. Virol..

[40] Xie X., Yang F. (2005). Interaction of white spot syndrome virus VP26 protein with actin. Virology.

[41] Zhang J.-Y., Liu Q.-H., Huang J. (2014). Multiple proteins of White spot syndrome virus involved in recognition of β-integrin. J. Biosci..

[42] Jiravanichpaisal P., Lee B.L., Söderhäll K. (2006). Cell-mediated immunity in arthropods: Hematopoiesis, coagulation, melanization and opsonization. Immunobiology.

[43] Whelan J. (1996). Selectin synthesis and inflammation. Trends Biochem. Sci..

[44] Johansson M.W., Söderhäll K. (1988). Isolation and purification of a cell adhesion factor from crayfish blood cells. J. Cell Biol..

[45] Pham L.N., Schneider D.S., Beckage N.E. (2008). Evidence for specificity and memory in the insect innate immune response. Insect Immunology.

[46] Cui C., Tang X., Xing J., Sheng X., Chi H., Zhan W. (2022). Single-cell RNA-seq uncovered hemocyte functional subtypes and their differentiational characteristics and connectivity with morphological subpopulations in *Litopenaeus vannamei*. Front. Immunol..

[47] Ng Y.S., Lee D.Y., Liu C.H., Tung C.Y., He S.T., Wang H.C. (2022). White Spot Syndrome Virus Triggers a Glycolytic Pathway in Shrimp Immune Cells (Hemocytes) to Benefit Its Replication. Front. Immunol..

