# Supplementary material for: A Novel Hemocyte-Specific Small Protein Participates in White Spot Syndrome Virus Infection via Binding to Viral Envelope Protein
Source: Viruses. 2023 Jan 13;15(1):227. doi: 10.3390/v15010227 (PMC9865939; doi:10.3390/v15010227)
Supplement: Supplementary file 1 [file viruses-15-00227-s001.zip › viruses-2131645-supplementary.pdf]

Supplementary Table S1. Primer sequences used in the functional study of LvHSSP genes

**Name**	**Sequence (5'-3')**
**cDNA cloning**	
LvHSSP-F	GGGAGTGTGTTTGTGGCACTG
LvHSSP-R	TCACTTGCCAGTCACAATCTG
**RT-PCR and qPCR**	
18S-qF	TATACGCTAGTGGAGCTGGAA
18S-qR	GGGGAGGTAGTGACGAAAAAT
LvHSSP-qF	GGCATCTAAGTTGGGCTAAG
LvHSSP-qR	CTCCGCAAAGGTTATGTTTT
VP28-qF	AAACCTCCGCATTCCTGTGA
VP28-qR	TCCGCATCTTCTTCCTTCAT
**RNAi**	
LvHSSP-dsF	TAATACGACTCACTATAGGGGCTTCACTGGGCTCTGAAG
LvHSSP-dsR	TAATACGACTCACTATAGGGCTTGCCAGTCACAATCTGTTTA
EGFP-dsF	TAATACGACTCACTATAGGGCAGTGCTTCAGCCGCTACCC
EGFP-dsR	TAATACGACTCACTATAGGGAGTTCACCTTGATGCCGTTCTT
**Protein expression**	
LvHSSP-pMAL-His-F	TCACATATGTCCATGGCTTCACTGGGCTCTGAAG
LvHSSP-pMAL-His-R	TACCTGCAGGGAATTCTCAGTGATGATGATGATGATGCTTGCCAGTCACAATCTGTTTA
**Mutant construction**	
HSSP^{WT}-GFP-F	CTTGGTACCGAGCTCGGATCCATGACGCGTGTGATGTTCC
HSSP^{WT}-GFP-R	GCCCTTGCTCACCATGGATCCCTTGCCAGTCACAATCTGTTTA
HSSP^{SPmut}-GFP-F1	GCGGTGGTCGCTGCTTTCGCCATCTGTGCCGAGTGGGCTT
HSSP^{SPmut}-GFP-F2	ATGACGCGTGTGATGTTCCTGGCGGTGGTCG
GFPmut-F	AGGAAGCTTGGTACCGAGCTCGGATCCATTGTTAGCAAGG
GFPmut-R	TGCTGGATATCTGCAGAATTCCACTTGTACAGCTCGTCCATG
ORF-GFPmut-F	CTTGGTACCGAGCTCGGATCCATGACGCGTGTGATGTTCC
ORF-GFPmut-R	GCCCTTGCTAACAATGGATCCCTTGCCAGTCACAATCTGTTTA
5'&ORF-GFPmut-F	CTTGGTACCGAGCTCGGATCCGGGAGTATGCTTGTGGCGCTG
5'&ORF-GFPmut-R	GCCCTTGCTAACAATGGATCCCTTGCCAGTCACAATCTGTTTA
5'&ORFmut-GFPmut-F	GCAGCCATTACGCGTGTGATGTTCCTG
5'&ORFmut-GFPmut-R	ACGCGTAATGGCTGCGTTGCTTCAGAA
**Co-Immunoprecipitation**	
pDHsp70-HSSP^{WSP}-F	CTTGGTACCGAGCTCGGATCCATGGCTTCACTGGGCTCTGAAG
pDHsp70-HSSP^{WSP}-R	TGCTGGATATCTGCAGAATTCCACTTGCCAGTCACAATCTGTTTA
pDHsp70-VP19-F	CTTGGTACCGAGCTCGGATCCATGGCCACCACGACTAAC
pDHsp70-VP19-R	TGCTGGATATCTGCAGAATTCCACTGCCTCCTCTTGGGGTA
pDHsp70-VP24-F	CTTGGTACCGAGCTCGGATCCATGCACATGTGGGGGGT

pDHsp70-VP24-R	TGCTGGATATCTGCAGAATTCCATTTTTCCCCAACCTTAAACAG
pDHsp70-VP26-F	CTTGGTACCGAGCTCGGATCCATGGAATTTGGCAACCTA
pDHsp70-VP26-R	TGCTGGATATCTGCAGAATTCCACTTCTTCTTGATTTCGTCCT
pDHsp70-VP28-F	CTTGGTACCGAGCTCGGATCCATGGATCTTTCTTTCACTCTT
pDHsp70-VP28-R	TGCTGGATATCTGCAGAATTCCACTCGGTCTCAGTGCCAG
